# Immunisation rates among children in Nuuk

**DOI:** 10.1080/22423982.2018.1426948

**Published:** 2018-02-02

**Authors:** Nadja Albertsen, Ivalu Meincke Fencker, Heidi Egede Noasen, Michael Lynge Pedersen

**Affiliations:** ^a^ Department of Medicine, Queen Ingrid Hospital, Nuuk, Greenland; ^b^ Queen Ingrid Health Care Centre, Nuuk, Greenland

**Keywords:** Vaccinations, immunisations, Arctic, WHO, Measles, Greenland, infectious diseases

## Abstract

The children immunisation programme in Greenland correlates to the one in Denmark with the addition of the Bacille Calmette–Guerin (BCG)-vaccine and the immunisation against Hepatitis B (HBV). The immunisation rate among children in Greenland has been and is currently unknown and this study aims to estimate the immunisation rates among children in Nuuk from 1 July 2015 until 30 June 2016. We did an observational cross-sectional study based on a statistical extraction identifying all children in Nuuk eligible for an immunization in the children immunisation programme from 1 July 2015 until 30 June 2016 and a review of their medical records. We found acceptable coverage rates among children younger than 12 months, but coverage rates lower than recommended by the World Health Organization (WHO) among older children. Among children between 15 months and 4 years the coverage dropped as low as 33.9 %. Increased awareness of child immunisation rates is suggested including continuously monitoring and adjusting of the organisation of the immunisation programme.

**Abbreviations:** Bacille Calmette–Guerin immunisation (BCG); Chief Medical Officer (CMO); Diphtheria, Tetanus, Pertussis, Polio, Haemophilus influenza B, Pneumococcal (DTP); Electronic medical report (EMR); Hepatitis B (HBV); Human Papilloma Virus (HPV); Measles, Mumps, Rubella (MMR); World Health Organization (WHO)

## Background

Children immunisations prevent infectious diseases from spreading, thus reducing illness, disability and death globally. The World Health Organization (WHO) aims towards a coverage rate of 90% nationwide and 80% in local districts [1], but low coverage is still a challenge and WHO estimates that 18.7 million infants worldwide are missing out on basic vaccines [2].

The children immunisation programme in Greenland is based on the children immunisation programme in Denmark, with the addition of the Bacille Calmette–Guerin (BCG) immunisation and the Hepatitis B (HBV) immunisation. The BCG-immunisation was introduced in 1949 due to a high incidence of tuberculosis and became a part of the children immunisation programme in 1955 [3]. The latest addition to the children immunisation programme in Greenland was in 2010, when the HBV- and Human Papilloma Virus (HPV)-immunisations were added [4].

In Greenland, immunisations in the children immunisation programme are free and all children with a permanent address in Greenland are eligible to enter the programme.

One earlier study found high immunisation coverage rates in Sisimiut from 1993 to 1998 among 596 children who were vaccinated before the age of 2 years. However, they found dropping rates for later immunisations. The coverage rates matched those in Western countries [5]. Another study from 2003 based on data from the Chief Medical Officer (CMO) from 2002 reported a high coverage countrywide regarding immunisations administered to children younger than two years, but with variations between districts [6]. However, these conclusions should be taken with serious reservation due to lack of possibility to validate data.

Until 2010, all immunisations were reported to the CMO on paper and not personalised, making it impossible for the CMO to validate if the data were registered correctly. After 2010, the immunisations in the children programme were no longer registered on paper but electronically and personalised, and a drop from 80 to 59% in the average coverage rate for all immunisations in the children programme nationwide was observed [7]. The CMO suspected faulty registration and because the registered data could not be validated, coverage rates have not been published by the CMO since 2011/2012 [8].

Currently, the immunisation rate among children, both in Nuuk and in Greenland in general, is unknown, but in March 2015 a new electronic medical report system (EMR) was introduced in Nuuk, including a registration module for immunisations. Since then, it has been possible to register administered immunisations in this system. Data can be extracted from the EMR system, allowing follow-up on immunisations and, given the importance of the immunisation programme, it is important validate the data-extraction as early as possible, in this case 18 months after the introduction of the EMR system.

Thus, the objective of this study is to estimate the immunisation rates among children in Nuuk from 1 July 2015 until 30 June 2016 based on data-extraction from the current EMR system in Nuuk.

## Materials and method

The study was designed as an observational cross-sectional study based on statistical extractions and review of medical records of children living in Nuuk at the time of study.

### Setting

Approximately 56,000 people live in Greenland [9].

Approximately 17,000 people live in the capital, Nuuk [10]. The birth rate of children in Nuuk is approximately 220 children each year [11].

Greenland consists of 16 towns and approximately 60 villages and is divided into five health care regions. There is a hospital or a health care centre in each town and Queen Ingrid’s Hospital in Nuuk is the central hospital of the country. Forty-eight of the villages have either a nurse station or a health care worker [12,13].

The healthcare system in Greenland is a publicly financed healthcare system, meaning that all treatment and immunisations in the children immunisation programme are free for anyone with a permanent address in Greenland. The programme contains vaccines against Tuberculosis, HBV, Diphtheria, Tetanus, Pertussis, Polio, Haemophilus Influenza type B, Streptococcus Pneumonia (the 13-valent immunisation), Measles, Mumps, Rubella and Human Papilloma Virus (see Table 1).

**Table 1.** The Greenlandic child immunisation program.

Recommended age at immunisation	Immunisation type
0 weeks (at birth)	BCG HBV
3 months	Di-Te-Per-Pol-Hib-HBV-Pneumococcal-1: *(Diphteria, Tetanus, Pertussis, Polio, Haemophilus influenza B, Hepatitis B, Pneumococcal (13-valent))*
5 months	Di-Te-Per-Pol-Hib-HBV-Pneumococcal-2
12 months	Di-Te-Per-Pol-Hib-HBV-Pneumococcal-3
15 months	MMR-1:*(Measles, Mumps, Rubella)*
4 years	MMR-2
5–6 years (preschool)	Di-Te-Per-Pol-Hib Boost
12 years	HPV (girls only) (0 + 6 mth) MMR-2* HBV (0 + 2 + 6mth) **

* for children older than 3 months per 1 September 2010 (ends in 2022) ** For children older than 4 years per 1 September 2010 (ends in 2018) The last changes of the program was made 1 October 2014 [8]

Immunisations are bought by the main pharmacy in Nuuk and distributed to the rest of the country from Nuuk. The number of immunisations used or discarded at the different health care centres are not recorded.

At birth, the BCG- and the HBV-vaccine are administered to the child by the attending midwife. Afterwards, a nurse monitors the development and health of the child until the child is eight or nine months old and the nurse has the possibility to remind the parents of the immunisations at three and five months. However, it is the parents’ responsibility to book the appointment at the local healthcare centre, where the immunisations are given [14]. When the child enters school, the immunisations are given to all children in specific school years by a nurse. Individual vaccine cards are not used in Greenland.

A new EMR system, Cambio COSMIC, was implemented in Nuuk 1 March 2015, including a registration module for immunisations in the children immunisations programme. Since 1 June 2015, it has also been possible to register vaccines given at birth.

### Study population

A statistical extraction identifying all children in Nuuk eligible for an immunisation in the children immunisation programme from 1 July 2015 till 30 June 2016 was performed and their immunisation status recorded.

The cohorts of children in Nuuk eligible for each of the immunisations given before the age of two were designed to ensure that all immunisations were registered in the EMR system unless the immunisation had been given with more than one month’s delay among the youngest in the group.

For the immunisations given at the age of four, the cohort was designed to ensure that all immunisations were registered in the EMR, unless the immunisation had been given with more than four months delay among the youngest.

For the immunisations administered in schools, the cohorts were designed to include children from the specific school years eligible for preschool-, HPV- and HBV immunisations.

The ages of the children regarding each immunisation are shown in Table 2.

**Table 2.** Overview of age group of included children for each immunisation.

Eligible for:	Born between	Age per 1 July 2016 (months)
0 years – BCG	1 July 2015 – 30 June 2016	0–12
0 years – HepB	1 July 2015 – 30 June 2016	0–12
3 months – HepB	1 March 2015 – 29 February 2016	4–16
3 months – Di-te-Ki-Pol-HiB-Pneu	1 March 2015 – 29 February 2016	4–16
5 months – HepB	1 January 2015 -31 December 2015	6–18
5 months – Di-te-Ki-Pol-HiB-Pneu	1 January 2015 -31 December 2015	6–18
12 months – HepB	1 June 2014 – 31 May 2015	13–25
12 months – Di-te-Ki-Pol-HiB-Pneu	1 June 2014 – 31 May 2015	13–25
15 months – MMR1	1 March 2014 – 28 February 2015	16–28
4 years – MMR2	1 March 2011 – 29 February 2012	52 – 64
5–6 years – Di-te-Ki-Pol-HiB	1 January 2009 – 31 December 2009	78–90
12 years – MMR2	1 January 2004 – 31 December 2004	138–150
12 years – HPV1	1 January 2003 – 31 December 2003	150–162
12 years – HPV2	1 January 2003 – 31 December 2003	150–162
12 years – HBV1	January 1 2003 – December 31 2003	150–162
12 years – HBV2	January 1 2003 – December 31 2003	150–162
12 years – HBV3	January 1 2003 – December 31 2003	150–162

All medical records of the children included in the cohorts were reviewed in order to validate the extracted immunisation status. The recorded status in the immunisation module was compared to the written entries and the registrations in the medication module in the EMR. If any discrepancy was found, the written entries and registrations in the medications module were considered to indicate the true immunisation status.

### Ethics

The ethics committee for medical research in Greenland approved the study.

## Results

### Immunisation coverage

The raw data extraction indicated that a total of 3511 immunisations should have been given in Nuuk during the study period in order to achieve a coverage rate of 100%. After review of the EMRs and the exclusion of stillborn, children who died before or during the study period and children living outside of Nuuk, the total number of immunisations that should have been administered during the study period was reduced to 3,260 (Table 3).

**Table 3.** Immunisation rates among included children in Nuuk Greenland.

		Extracted data (raw) Number of children			Data after review (validated) Number of children			
Age	Immunisation type	Vaccinated	Not vaccinated	Total	Vaccinated	Not vaccinated	Total	Coverage rate (%)
0 months	BCG	226	11		227	6	233	97.4
0 months	Hepatitis B	230	7		230	3	233	98.7
3 months	Di-Te-Per-Pol-Hib- Pneumococcal-1	210	31		215	20	235	91.5
3 months	Hepatitis B	210	31		215	20	235	91.5
5 months	Di-Te-Per-Pol-Hib- Pneumococcal-2	179	57		178	44	222	80.2
5 months	Hepatitis B	180	56		179	43	222	80.6
12 months	Di-Te-Per-Pol-Hib- Pneumococcal-3	118	67		120	52	172	69.8
12 months	Hepatitis B	118	67		120	52	172	69.8
15 months	MMR-1	85	86		88	71	159	55.3
4 years	MMR-2	59	137		59	115	174	33.9
5–6 years (Preschool)	Di-Te-Per-Pol-Hib-boost	134	92		142	66	208	68.3
12 years	MMR-2	174	42		188	18	206	91.3
12 years (girls only)	HPV-1	71	46		68	26	94	72.3
12 years (girls only)	HPV-2	40	78		41	54	95	43.2
12 years	Hepatitis B 1	166	57		165	35	200	82.5
12 years	Hepatitis B 2	121	102		142	58	200	71.0
12 years	Hepatitis B 3	100	123		99	101	200	49.5
**Total**		**2421**	**1090**	**3511**	**2476**	**784**	**3260**	

The raw data extraction found the total number of vaccinated children to be 2421, but after the review of the children’s EMR, this number was adjusted to 2476. The initially lower number was due to faulty registration in the immunisation module in COSMIC.

The immunisation coverage rate of the newborn was the highest found in the study (98.7% for HBV and 97.4% for BCG). The coverage rate for children younger than 12-months old (not including BCG or HBV at 0 months) was also high (80.2–91.5%). At 12 months the coverage rate dropped to 69.8% and for children between 15 months and 4 years the coverage dropped as low as 33.9% for MMR-2.

For preschool-children the coverage rate improved, but was still low at 68.3%.

A further improvement in coverage rate was found for MMR-2 (91.3%), HPV-1 (72.3%) and HBV 1 (82.5%) among the 12-year old children. However, the rate dropped to 43.2% for HPV-2 and 71.0% for HBV 2 and 49.5 for HBV 3, which can be a result of a delay in the vaccination programme in one school in Nuuk.

The coverage rates of the immunisations can be seen in Table 3.

## Discussion

In short, we found the highest immunisation coverage among the newborn and the children younger than one year and the 12-year old children who are immunised in school. This could indicate that close contact to the health system is needed to ensure that the immunisations are given.

WHO recommends a high coverage rate of immunisations in the Children Immunisation Programme in order to protect the population against infectious diseases such as Polio and Measles. The target coverage rate for measles, according to the WHO, is 95% [15] and our study found a coverage rate in Nuuk as low as 55.3% at 15 months and 33.9% at four years, meaning that an outbreak of measles could become a general health threat in Nuuk and possibly the rest of Greenland.

Regarding Polio, WHO recommends a coverage rate of at least 90% [15]. This was only achieved at three months where we found a coverage rate of DTP (Diphtheria, Tetanus, Pertussis, Polio, Haemophilus Influenza type B, Streptococcus Pneumonia) of 91.5%. The DTP-immunisation given at three, five and 12 months protects the child until the age of five or six, but the booster immunisation gives the child lifelong protection against Polio. At five months and 12 months we found coverage rates of 80.2 and 69.8%, which also shows that the coverage rate drops the older the child gets. One of the explanations for this could be that the family is no longer in direct contact to a nurse, and therefore not reminded of immunisations, when the child is older than eight months. However, the coverage rate for the DTP-booster at preschool, which is administered by a school-nurse and given in connection to a general health check, was 68.3% in our study which is almost the same as at 12 months. But, even though the coverage rate of DTP doesn’t increase at preschool level, the coverage rate is an improvement when compared to the coverage rate of MMR (Measles, Mumps, Rubella) at 15 months and four years, indicating that some of the children that have fallen out of the Immunisation Programme, are being picked up again when entering school.

We found coverage rates above 97% of the BCG- and the HBV-immunisation given at birth, which reflects a well-functioning system regarding these two immunisations and a willingness among parents to protect their children against Hepatitis B and severe Tuberculosis. Hepatitis B is an endemic disease in Greenland with around eight % of the population being chronically infected. Screening of pregnant women was introduced in 1992 and the immunisation of newborn was introduced in 2010 [16]. The goal for WHO is a national coverage of 90% in 2020 [1]. The immunisation against Hepatitis B is given in conjunction with DTP at age three, five and 12 months and given as a single immunisation only at birth and at 12 years. As mentioned above, the coverage rate at birth is high (98.7%), but at 12 years the coverage rate is 82.5%, which is lower than the coverage rate recommended by WHO, meaning that children born before 2010 has a high risk of getting infected at some point in their lives. It should be noted that at age 12 years, three doses are administered during a 6-month period and the lower coverage rates for dose two and three at 12 years found in this study could be due to some children having only received the first dose and the rest are planned later this year.

In Denmark, the coverage rate of Children Immunisation Programme in 2015 was higher than in Greenland, but below the WHO recommendation when regarding Measles. The coverage rate of MMR-1 and MMR-2 was 88% and 83% and the coverage rate of DTP was 93% at three months, 90% at five months and 90% at 12 months [15]. In another arctic region, Alaska, the coverage rates of MMR-1 and MMR-2 in 2014 were 90.2% and 92.7%, thus reaching the WHO recommendation. Regarding DTP, a coverage rate of 92.7% was reached [17].

The low coverage rates of immunisations given after early childhood in Nuuk may have different causes. In Denmark, all immunisations given in the programme are registered centrally and the data is available to health care professionals making it easy to follow a child’s immunisation status. Furthermore, reminders are sent to parents to children aged two, six and a half or 14 years if they have already missed one or more immunisations in the programme [15].

In Alaska, the immunisations are also registered centrally and it is required by law that children entering public or private school are immunised unless they are exempted due to medical or religious reasons [18].

A review found several factors are associated with suboptimal compliance to the children’s immunisation programme in developed countries [19]. They divide the factors into two different categories; parental-childhood characteristics and healthcare structure/health care professional characteristics. They find such characteristics as mothers younger than 24 years, large family size, late birth order, low socio-economic status, low income, lack of knowledge regarding diseases/immunisations, inadequate support from healthcare structures and lack of accessibility to healthcare centres associated with low immunisation rates. Some of these factors are found in Greenland, where the average age of first-time mothers is 24.7 years [20] and the fertility rate is the second highest in the Nordic countries.

Lack of knowledge about the importance of immunisations, contraindications and side-effects may play a part. A study from the US [21] showed that one of the main reasons for low coverage was vaccine-hesitancy among parents and that medical professionals lacked the knowledge to provide the parents with the correct and needed information and might even have doubts about the immunisations themselves. This study argues that medical professionals should become better at handling the parents’ doubts and fears and that a more paternalistic approach towards parents might be needed regarding immunisations.

Another study [22] found that once a child’s immunisations were delayed, not only was the risk of the child getting infected or becoming a vector for infection higher, the risk of the following immunisations also being delayed or not given at all, was higher than among children being vaccinated on time. They found that making immunisations more available, providing reminders and using topical analgesics might improve the number of children being vaccinated on time and completing the entire immunisation programme.

Different strategies to improve immunisation rates can be considered. A reminder-system using local health care workers and SMS has proven useful among Australian Aboriginals [23]. Other electronic tools such as apps for the parents could be considered, or organising events where multiple children are immunised and information accessible for the parents or giving more immunisations at the same age, so that the risk of falling behind is lower. Also, in Nuuk, the immunisations are given by a nurse that the child or parent may not have met beforehand and no topical analgesia is used. One can imagine that this could make the experience an uncomfortable and traumatic one, and the risk of the parents not wanting their child to receive more immunisations higher.

The above speculations highlight a need for more specific studies focusing on the access to and considerations for and against immunisations in Greenland. And as Cambio COSMIC will be implemented in the rest of Greenland, data extraction and estimations of immunisation coverage rates in all of Greenland will be possible within the coming years, making as estimation of the true scope of the problem possible.

### Strengths and limitations

Strengths: this is the first study of the coverage rate in the Children Immunisation Programme in Nuuk with the possibility to extract personalised data and therefore also the possibility to validate the data.

Limitations: the current EMR system in Nuuk was implemented in 2015 and has yet to be taken into full use in the rest of the country, making it impossible to extract data from other towns or to extract data from before June 2015. This means that the data are limited and some of the cohorts small.

Regarding coverage rates, the rates found in this study might be lower than the actual coverage rates, as some of the children in this study has moved to Nuuk from other towns and their previous immunisation status is unknown, making them appear as not having had their immunisations. Other children might have experienced delays in the immunisation programme and may also appear as not having had their immunisations, making the coverage rate appear lower than it actually is.

## Conclusion

The coverage rates of immunisations in the Children Immunisation Programme among children in Nuuk are lower than the recommended coverage rates by WHO, except among the newborn. The coverage rates in the rest of Greenland remain unknown, but should be explored when it is possible to extract data from other areas than Nuuk and the coverage rates should be closely monitored in the future in order to ensure better protection against vaccine-preventable diseases in Greenland.

Studies focusing on the reasons why the coverage rate is low in Nuuk, and possibly the rest of Greenland, are recommended.
